# On-Ground Vineyard Reconstruction Using a LiDAR-Based Automated System

**DOI:** 10.3390/s20041102

**Published:** 2020-02-18

**Authors:** Hugo Moreno, Constantino Valero, José María Bengochea-Guevara, Ángela Ribeiro, Miguel Garrido-Izard, Dionisio Andújar

**Affiliations:** 1 Centre for Automation and Robotics, CAR-CSIC, Arganda del Rey, 28500 Madrid, Spain; hugo.moreno.parrizas@csic.es (H.M.); jose.bengochea@csic.es (J.M.B.-G.); angela.ribeiro@csic.es (Á.R.); 2 Laboratorio de Propiedades Físicas (LPF_TRAGRALIA), ETSIAAB, Universidad Politécnica de Madrid, 28040 Madrid, Spain; constantino.valero@upm.es (C.V.); miguel.garrido.izard@upm.es (M.G.-I.)

**Keywords:** laser measurements, plant volume estimation, vineyard proximal sensing, vineshoot volume

## Abstract

Crop 3D modeling allows site-specific management at different crop stages. In recent years, light detection and ranging (LiDAR) sensors have been widely used for gathering information about plant architecture to extract biophysical parameters for decision-making programs. The study reconstructed vineyard crops using light detection and ranging (LiDAR) technology. Its accuracy and performance were assessed for vineyard crop characterization using distance measurements, aiming to obtain a 3D reconstruction. A LiDAR sensor was installed on-board a mobile platform equipped with an RTK-GNSS receiver for crop 2D scanning. The LiDAR system consisted of a 2D time-of-flight sensor, a gimbal connecting the device to the structure, and an RTK-GPS to record the sensor data position. The LiDAR sensor was facing downwards installed on-board an electric platform. It scans in planes perpendicular to the travel direction. Measurements of distance between the LiDAR and the vineyards had a high spatial resolution, providing high-density 3D point clouds. The 3D point cloud was obtained containing all the points where the laser beam impacted. The fusion of LiDAR impacts and the positions of each associated to the RTK-GPS allowed the creation of the 3D structure. Although point clouds were already filtered, discarding points out of the study area, the branch volume cannot be directly calculated, since it turns into a 3D solid cluster that encloses a volume. To obtain the 3D object surface, and therefore to be able to calculate the volume enclosed by this surface, a suitable alpha shape was generated as an outline that envelops the outer points of the point cloud. The 3D scenes were obtained during the winter season when only branches were present and defoliated. The models were used to extract information related to height and branch volume. These models might be used for automatic pruning or relating this parameter to evaluate the future yield at each location. The 3D map was correlated with ground truth, which was manually determined, pruning the remaining weight. The number of scans by LiDAR influenced the relationship with the actual biomass measurements and had a significant effect on the treatments. A positive linear fit was obtained for the comparison between actual dry biomass and LiDAR volume. The influence of individual treatments was of low significance. The results showed strong correlations with actual values of biomass and volume with R2 = 0.75, and when comparing LiDAR scans with weight, the R2 rose up to 0.85. The obtained values show that this LiDAR technique is also valid for branch reconstruction with great advantages over other types of non-contact ranging sensors, regarding a high sampling resolution and high sampling rates. Even narrow branches were properly detected, which demonstrates the accuracy of the system working on difficult scenarios such as defoliated crops.

## 1. Introduction

Nowadays, agriculture features much more in terms of economy than it did in the past, since food drives the world where access to adequate food is the primary concern. The exponential growth of the world’s population in the coming years poses an increased pressure to global food production systems. The global population is expected to increase from 7.6 to 10.5 billion people by 2067, while the arable land per capita will decrease by 25% [1]. Moreover, a major ongoing concern in the preservation of the environment leads to a more sustainable agriculture. At this critical juncture, Precision Agriculture (PA) has to deal with the current and future agriculture market as it increasingly demands improved productivity and efficiency together with the goals of saving inputs, higher yields, cutting costs, saving time, and making it more profitable [2]. Thus, PA plays a key role for these purposes. Farming management strategies and a site-specific treatment are needed to help farmers on the grounds of decision-making policies to enhance production while being sustainable [3]. In this scenario, a detailed characterization of plant structure leads to an optimum management of crops. Crop phenotyping is of high importance to assess the geometric characterization of vegetation since it is closely linked to growth status and productivity [4]. A thorough knowledge of plant architecture allows an adequate application of pesticides, fertilizers, and water inputs; hence, it helps to reduce costs and to obtain a higher crop yield. 

Crop characterization can be done either manually or semi-automatically with the help of PA. In this context, there is a slow but gradual adoption of the extensive use of PA to the detriment of manual measurements, since they are time-consuming, tedious, and often lead to inaccurate processes [5]; hence, they are not economically viable to sample large crop areas. Light detection and ranging (LiDAR) or other distance-measurement systems can be easily incorporated into current machinery or tractors in order to scan the field while doing other task such as tillage or fertilization. The use of a monitoring platform (such as this research work) should not be necessary for a transition to real field applications. This would reduce the cost, since a specific monitoring would not be required. In addition, the use of LiDAR information is easy and fast to process, and it could be incorporated to Precision Farming applications in a short period of time. Many approaches have been already presented [1]. Although the balance between cost/definition needs to be explored, some applications based on branch volume require definition. However, if that definition is reached in a fast and reliable manner, the systems could be used on real farming practices. Nevertheless, robotic automation is focused on optimizing workflow, minimizing manual labor, and producing more reliable information from crops along with the ability of analyzing the spatial variability of the extracted parameters (such as leaf area index, crop height, or volume). Moreover, sustainable agriculture demands variable rate technologies to properly respond to inter and intra-field variability in crops, as they are heterogeneous and irregular. This advances toward site-specific management, reducing inputs (along with the impact of over-application on the environment), minimizing labor costs, and maximizing productivity. PA uses a wide variety of sensors to obtain geometric three-dimensional (3D) reconstructions of crops along with other distinctive architectural features. RGB cameras have been widely used to assess phenotypic traits for several purposes. Guan et al. 2018 estimated with accuracy soybean height. An image-based projected area helps to quantify shoot biomass and diameter as phenotype parameters in weeds [6]. Naik, Zhang [7] developed an automatic assessment of iron deficiency chlorosis based on canopy trait features using a smartphone app for rapid and real time severity rating in the field. Yamamoto, Guo [8] using a fixed-point camera calculated the yield estimation of intact tomato fruits, including mature, immature, and young fruits with remarkable results. There is a wide variety of studies using camera-based systems for plant characterization. Although RGB cameras are inexpensive and quick to operate, along with presenting an easy processing system, they are not of high resolution for 3D modeling in planar mode. The need for an efficient and automatic phenotyping of traits across large populations is still a challenge. Given this scenario, ultrasonic and LiDAR (light detection and ranging) sensors have featured prominently over the past years, as they are adequate for rapid data acquisition when mounted on a vehicle. Ultrasonic sensors have been used for the geometrical characterization of plants and weed detection [9,10]. However, LiDAR sensors compared to ultrasonic sensors when attached to mobile platforms provide a higher 3D spatial resolution and measuring speed; i.e., it collects more data into each reading, and it has a broader field of view [11,12,13,14]. 

LiDAR sensors have made a significant contribution to plant structure characterization and crop modeling. Key geometrical features such as the leaf area index, crop height or canopy volume, and plant biomass are directly related to health status and plant vigor; in this manner, unforeseen diseases can be indirectly detected in early stages. In this regard, there are many studies being conducted with LiDAR sensors, as it receives widespread support among researchers as a contactless, non-destructive, and relatively affordable technique for multiple and different purposes. Andújar, Escolà [15] used a tripod-mounted LiDAR to detect and discriminate weeds using distance and reflectance values. Martínez-Guanter, Garrido-Izard [16] combined 2D LiDAR measurements with the tractor movement to determine plant spacing for physical or chemical weed control on row crops. 3D maize plant reconstruction to support plant phenotyping and precision agriculture processes was proposed by Garrido, Paraforos [17]. On-ground applications mainly focus on tree canopy geometric characterization for estimating canopy volume and height [18] as key factors for site-specific management and implementing precision agriculture techniques. To assess improvements in the operating conditions of harvesters in order to minimize fruit damage in high-density olive groves. Pérez-Ruiz, Rallo [19] attached two LiDAR sensing devices to the front of a tractor to estimate volume and biomass losses during harvesting. Ref. [4] characterized poplar trees for biomass production, obtaining strong plant height and volume relationships with LiDAR measurements. Li, Dai [20] developed a system to estimate leaf area index and density (LAI and LAD respectively) to gain a better insight into Magnolia trees photosynthesis. A ground-based mechatronic LiDAR was utilized for porosity and crown surface estimation in several tree species as two important parameters in the characterization of crops for crop monitoring and precision agriculture tasks. Jimenez-Berni, Deery [21] mounted a LiDAR along with other sensors to assess canopy height, ground cover, and above-ground biomass using a mobile platform with the aim of improving genotypes and agronomic interventions.

The 2D LiDAR sensor technology allows 3D scanning of all types of objects when being displaced perpendicular to crop rows. This methodology was also used for the geometric characterization of vineyards [22]. The authors proved that proper reconstruction can be achieved with the use of a LiDAR sensor. Those measurements can be used for precision spraying based on the information provided by the sensor. The results proved that this method improves the application of agrochemicals or pesticides. In addition, the distance measurement can also be related to the drift while applying agrochemicals. The system measures the distance between the sensor and the objects around, with a large spatial density of points (resolution) and a very high frequency (thousands of points per second). Two-dimensional (2D) (x,y) and 3D cloud points (x,y,z) can be obtained by applying appropriate algorithms, which enable the reconstruction and description of the geometrical structure of the plants with high precision. The use of this sensor to create a point cloud corresponding to a plane with the associated position of each reading makes this device an appropriate tool to assess the crop structure in a digital format of massive data point clouds. Moreover, LiDAR sensors can measure the reflection value, which is a reflectivity index. This information could be used consistently in addition to distances to define density features [15]. Since there is widespread support for LiDAR systems due to their high speed of measurement, resolution, and accuracy, in vineyards, they also can be used for 3D crop models to assess vegetative volume and other key geometric features such as the LAI or LAD [22]. However, these phenotypic characteristics are not solely responsible for the development and health status of a plant; assessing wood volume and specifically vineshoot volume can be taken into consideration as well for gaining a better insight into the vines’ development processes. 

In this paper, an RTK-GNSS system and a 2D LiDAR sensor were mounted on a mobile platform to characterize the geometrical attributes of vineyards. The main objective of this study was to map large crop areas in order to evaluate the structural characterization of vines. Moreover, this paper aims to assess phenotypic traits such as height and vineshoot volume as surrogates of plant dry biomass. In this way, a proper 3D model of crop biophysical parameters would allow to implement techniques such as automated pruning, irrigation programs, and variable rate technology.

## 2. Materials and Methods

### 2.1. Site Location

Field measurements were conducted in a vineyard field located in central Spain on the experimental station of “El Socorro” (IMIDRA, Colmenar de Oreja, Madrid, Spain). Experimental data were obtained during the winter–spring season of 2018 at several stages. Measurements were carried out in the vineyards of the Experimental Station on 25 January. The site (40°8′ N, 3°22′ W; altitude 750 m a.s.l.) is characterized by 436 mm mean annual precipitation and a mean temperature of 13.5 °C. Vines were planted at an inter-row distance of 2.5 m. 

The experiment was carried out in a 2 ha vineyard field. The field was divided into five different treatments with three plots per treatment. Each plot was formed by 10 vines. In total, 150 vines were cut and analyzed separately. The vineyard plot was segmented into 10-vine batches according to different ways of cultivating. This implies a wide variety of tillage systems, fertilizing and pesticide treatments, and pruning techniques that have the potential to conserve soil and water by reducing their loss along with increasing the crop yield. These ways of cultivating are aimed at preserving and improving the physical and chemical characteristics of the soil together whilst improving plant production. In this manner, the area studied was divided into five different cultivating techniques, and each of them features three rows, i.e., replications, in different locations within the same vineyard. (A) It is defined as intensive with chemical treatment. It aims to maximize yield (S1). (B) It is considered a low-input or mixed technique, since it combines treatments with pesticides (herbicides and fungicides) and mechanical work, which ends up having a lower yield (S2). (C) It is based on an intensive mechanical weed control (using an in-row weeder). This system rotates the vegetal cover every three years. The last rotation began in 2015, at the beginning of the series under study (S3_1 and S3_2). (D) Harrowing is done in the inter-row areas (S4_1). (E) A vegetal cover is left in the inter-row areas (S4_2). The pruning of the areas under study was carried out on 20 and 21 February. Immediately, the pruning branches were taken to the laboratory for dry biomass determination. The dry branches were weighed manually within the vineyard and after the drier treatment. Branches were characterized for a diameter lower than 1 cm. Thus, the end details of branches were sometimes missing due to their low diameter. Thus, an underestimation of the real branch volume was observed. This effect was mainly observed on those parts of branches with a diameter of less than 2 mm. 

### 2.2. Sampling System

**Figure 1:**
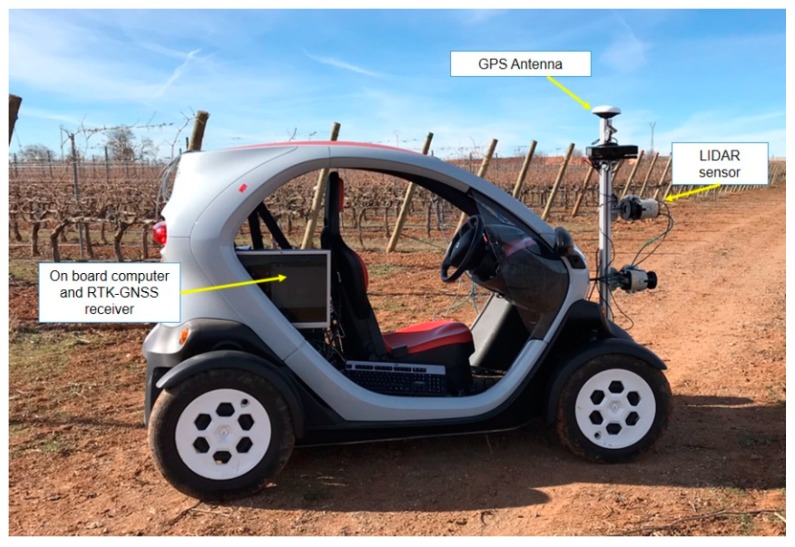
Electric mobile platform comprising light detection and ranging (LiDAR) laser scanner and GPS-RTK receiver mounted on a lightweight extruded aluminum with adjustable height. Data is captured on an on-board computer.

Readings were taken using a mobile platform based on a Twizy Urban 80 model (Renault), which is a plug-in electric vehicle. Sensors were installed on a height-adjustable instrument boom attached to the front of the platform (Figure 1). This mobile platform allows very low speeds, below 3 km/h^−1^, which is a key factor to acquire high-quality information, since data resolution is significantly affected by the speed of the mobile platform. The mobile platform comprised the following instrumentation:

(1) A general-purpose laser scanner (model LMS-111; SICK AG, Waldkirch, Germany) was used for the measurement system. A gimbal was installed to dynamically stabilize the LiDAR. The sensors had an operating range: from 0.5 to 20 m LiDAR Class: 1 (IEC 60825-1) with an angular resolution of 0.5″. The statistical error is 12 mm, and the light spot size at optics cover/18 m: 8 mm/300 mm. The measurement principle is based on a 2D divergent laser scanner with a maximum scanning angle of 270 with a selectable lateral resolution of between 0.25° and 0.50°. During this process, a laser pulse and therefore a measurement is triggered after an angular step of 0.25° or 0.50°. The emitted laser beams are deflected using a mirror and scan the surroundings in a circular manner. The measurements are triggered at regular angular steps using an angular encoder. The accuracy of the device is ±30 mm in a single-shot measurement and a 12 mm standard deviation (SICK AG 2008). The frequency was set up at 25 Hz. The source of light is a pulsed infrared laser of 905 nm. The distance between the laser scanner and the object of interest was determined according to the time-of-flight method by measuring the time interval between an outgoing laser pulse and the return signal reflected by the target object. By distance extraction, a 3D point cloud was obtained containing all the points where the laser beam impacted. The point cloud model was a composition of vertical slices of the vineyard reconstruction. The distance between consecutives planes of scan was about 10 ± 2 mm depending the platform speed. The fusion of LiDAR impacts and the positions of each associated to the RTK-GPS allowed the creation of the 3D structure. For each scan, the points that intersected the vineyard created a plane corresponding to a full 180° LiDAR scan. The sensor was located at a height of 1.20 cm above the ground level and laterally (facing sideways) pointing perpendicularly toward the vineyard rows (at a distance of approximately 1 m from the crop row).
(2) An RTK-GPS receiver (R220, Hemisphere, Scottsdale, AZ, USA) was also mounted in the vehicle on the top of the aluminum structure and aligned with the center of the sensor. It can provide geospatial position according to the NMEA (National Marine Electronics Association) standard at a 20 Hz sample rate with an accuracy capability of 20 mm precision + 2 ppm (in kinematic mode).
(3) An on-board computer (Intel Core i7-4771@3.5 GHz processor, 16 GB of RAM, and a NVIDIA GeForce GTX 660 graphic card) was used to register sensors readings, which were transferred via Ethernet communication port.

The LiDAR was installed to obtain the lateral projection of the vines because it provided the best information on the structure of the vines. Depth data was collected by setting the field of view to 270° at 0.5° angular steps and a sampling frequency of 50 Hz (every 20 ms). LiDAR readings were acquired and processed by a developed LabVIEW programme (National Instruments Co., Austin, TX, USA) [19]. Height profiles of the vines were obtained by distance extraction, generating a high-density point cloud containing all points where the laser beam impacted. The vehicle traveled in a straight line parallel to the row through the center of the inter-row area, so the raw data consisted of vertical slices of the vine surface. In this way, each cross-section comprised the intersection points between the laser beam and the vine. The distance between slices was about 5 mm.

Three-dimensional (3D) points are possible since the 2D LiDAR is displaced along the rows, and vertical transects are made up of beam impacts on the each side of the crop row. Thereby, by scanning, the transversal sections’ branch volume referred to pruning remains is estimated, and it is later related to dry branch biomass. Therefore, the RTK-GNSS receiver was set at 10 Hz to integrate the LiDAR raw data into a local system to obtain a 3D structure of the vines. Since the mobile platform traveled in the direction of the *X*-axis (parallel to the vineyard rows), a Cartesian coordinate system was set by projecting the vine cross-sections along the *Y*-axis (in the direction of the crop depth) and *Z*-axis (in the direction of the crop growth). In this manner, the LiDAR sensor is the origin of the local coordinate system, and every scan had a distinct GNSS position; therefore, a 3D point cloud is generated though the LiDAR is a 2D laser scanner. The data were saved separately for each vineyard row and area of study.

### 2.3. Data Processing

LiDAR raw data was pre-processed using the RTK-GNSS receiver information for the discretization of the X-axis (direction of movement). In this way, vertical slices (cross-sections of the vines) of the areas studied were obtained within the ZY-plane, calculated branch volume, and compared to ground truth-values (plant dry biomass). It was necessary to pre-process the data as a prerequisite for calculating branch volume, since LiDAR densely sample the structure of the vines, producing massive datasets along with the high complexity of its data. LiDAR data provide a 3D representation of the vine canopy, which can be processed in multiples forms, offering a wide range of measurements from the same original raw data. Key traits that are relevant in phenotyping and plant physiological structure: ground cover and above-ground biomass (focusing on vine shoot volume) were extracted. The pre-process of the voluminous LIDAR raw data was performed using a Matlab R2017b (MathWorks, Natick, MA, USA) algorithm described in [16]. Laser scans were converted from polar coordinates (angles and distances) to rectangular (Cartesian) coordinates using a horizontal reference direction. For this purpose, data were transformed according to the angles of roll, pitch, and yaw to integrate the scans from LiDAR into the mobile platform coordinate system. After applying a 3D affine transformation, next, a translation was performed by shearing along over the X-axis (the direction of movement) for the actual LiDAR orientation. For this purpose, RTK-GNSS data and LiDAR data were time-stamped to adjust the actual orientation of the LiDAR. According to this approach, the local LiDAR point cloud was converted into a relative coordinate system, since NMEA data from the RTK-GNSS were used as an encoder for the vehicle advance. Therefore, homogeneous coordinates were obtained for the subsequent 3D calculations. 

**Figure 2:**
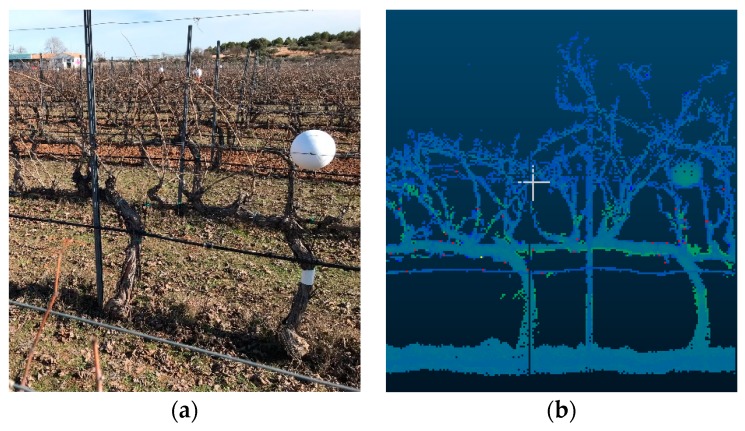
Fixed physical references (white sphere) were placed within the row to delimit the studied area (**a**). Section cloud points projected onto Y-axis (the depth of the crop). (**b**) The horizontal line depicts the XY-plane cross-section (with regard to the direction of movement).

To properly obtain branch (vine shoot) volume, it is fundamental to accurately represent the 3D point cloud in order to assess its key geometric features. The output data from MATLAB can be visualized in the form of a 3D point cloud using Cloud Compare (CloudCompare 2.9.1 GNU License, Paris, France). The data comprise a matrix whose dimension is three columns (x, y, and z) and n rows where n is the number of scans. The scanned rows were marked out with physical references to assess and extract the geometrical features of 10-vine batches (Figure 2a). In order to eliminate those points that did not belong to the vine shoots (either they appear isolated or represent the stump, cordon, or the soil), they were delimited in the point cloud representation by manually projecting a cross-section (within the XY-plane) set at the average height of the buds (Figure 2b). Training structures were later removed by subtracting their volume from the totality (horizontal wires were discarded as they have a negligible effect in the volume calculations). Finally, data points that were considered outliers (those whose average distance to its 64 neighbors is greater than the standard deviation of the distance to the neighbors of all the points) were removed automatically from the point cloud using [23]. After filtering, the output data represented only the vine shoot geometry for every 10-vine batch. 

**Figure 3:**
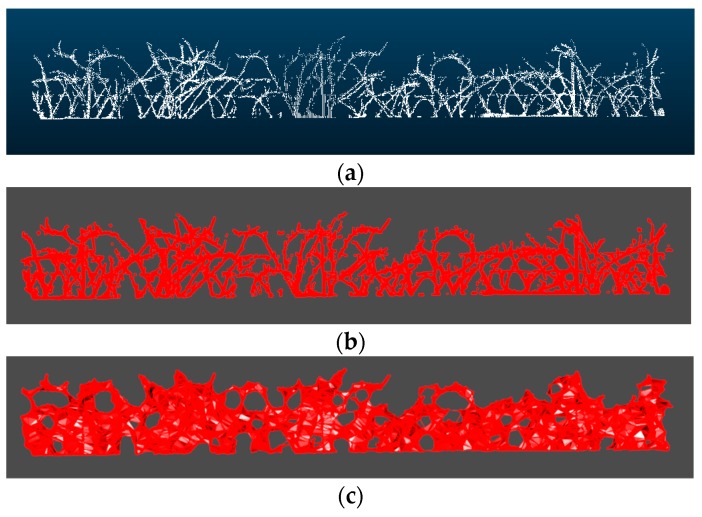

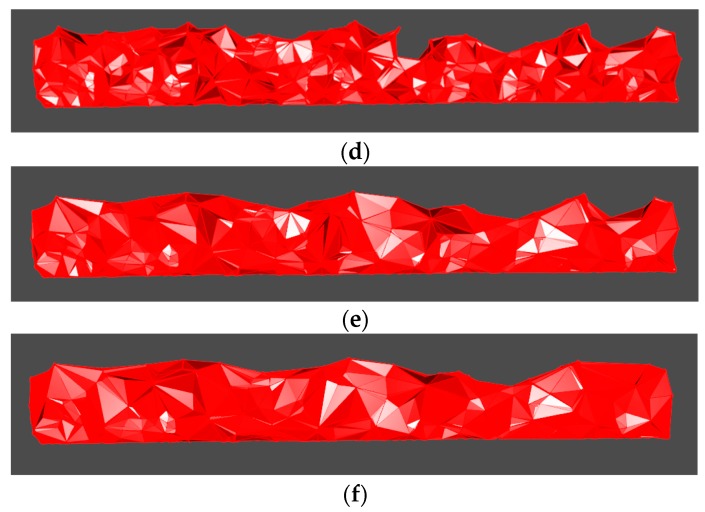
(**a**) Scanned point cloud of the branches of a section, (**b**) Alpha shape of the point cloud showed in (a) with alpha = 0.1, (**c**) with alpha = 0.3, (**d**) with alpha = 0.5, (**e**) with alpha = 0.7, and (**f**) with alpha = 0.9.

The resulting point clouds representing the vine shoots were strictly woody structures, since at that maturity stage, there were no leaves due to the winter season. Although point clouds were already filtered, discarding points out of the study area, branch volume cannot be directly calculated since it turns into a 3D solid clusters that encloses a volume (Figure 3). For that purpose, the alpha-shape (α-shape onwards) algorithm [24] was employed, as it had a proper performance in other studies in agriculture [14,25,26].To obtain the 3D object surface, and therefore, to be able to calculate the volume enclosed by this surface, a suitable alpha shape was generated as an outline that envelops the outer points of the point cloud. The degree of fit is defined by the index α. The more the α value decreases, the tighter the outline fits the points, producing a smaller surface to enclose a set of 3D points (loose shapes correspond to high α values). The most suitable α index would be the one that encloses the smallest volume whilst maintaining a solid surface free from voids. To calculate the α-shape of the branches of each section, thus to compute the volume enclosed, a 3D algorithm available in the alphashape3d package from R [27] based on the original work of [24] was used.

**Figure 4:**
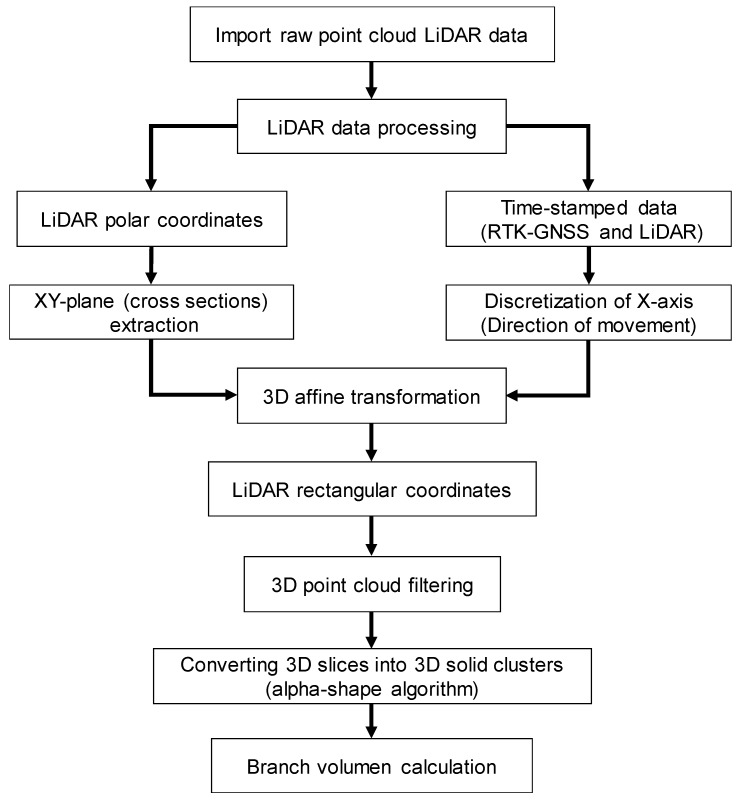
Data processing block diagram.

The choice of a proper value for α is completely decisive for the volume estimation. Several tests with different values of α were performed, finding that the α-shape generated using an α of 0.1 was the most faithful to the real outline of the branches. Examples of the α-shapes generated with different values of α for the same point cloud section can be seen in Figure 3. The entire process can be seen at Figure 4.

### 2.4. Statistical Analysis

Regression analyses were performed to identify the potential abilities of the system proposed to quantify the weight of the pruning remains. Therefore, the output data acquired from CloudCompare were processed statistically to assess the capabilities of the system for crop phenotyping, hence the key geometrical features for this study, i.e., vine shoots volume. In this manner, the actual parameters of pruning remains (ground truth) were weighted in order to be compared with the structural parameters coming from the 3D models created. A linear approach was applied to model the geometrical reliability of the LiDAR for estimating branch volume and hence dried biomass. ANOVA was applied to the number of scans and volumes assessed with the LiDAR to test their effect on the vineyard shoot weights, i.e., to identify the relation between the LiDAR readings and the actual parameters of volume and dry biomass. Further regression analyses were performed in order to determine the correspondence of structural parameters from the 3D models and the actual data measured on the field, thereby testing the crop phenotyping capabilities of the system. For this purpose, the LiDAR scans and output volume data from CloudCompare were contrasted with the vineyard shoot weights (dry biomass) and fitting linear models to all data, as well as to data by treatment. 

## 3. Results and Discussion

**Figure 5:**
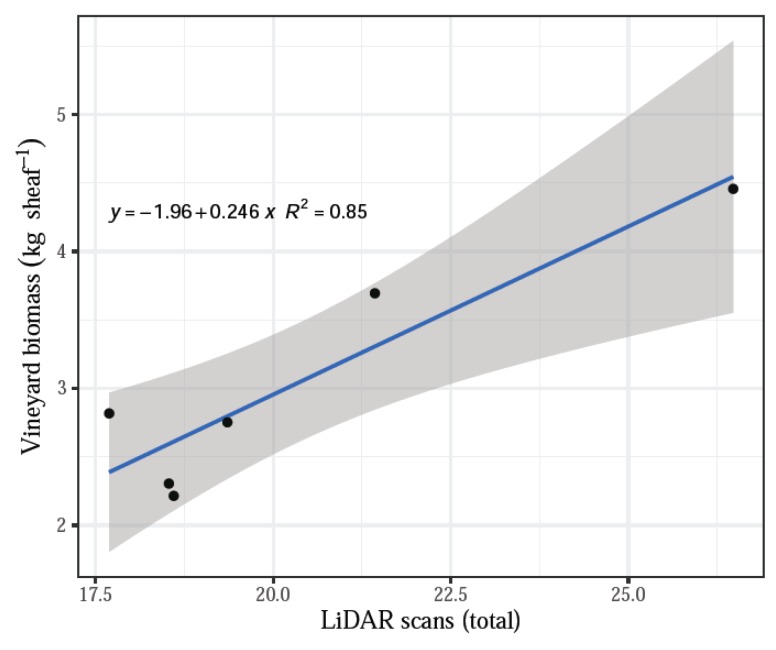
LiDAR scans versus weight (vineyard biomass).

**Figure 6:**
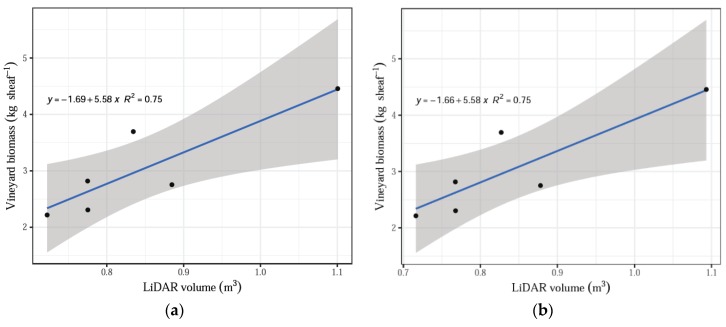
(**a**) LiDAR volume versus average value for vineyard biomass. (**b**) LiDAR volume without training structures versus average value for biomass.

The number of scans by LiDAR influenced the relationship with the actual biomass measurements and had a significant effect on treatments. The remaining parameters assessed by the system, namely, shoot volume and shoot volume without the tutoring prob, did not have a significant effect when contrasted with the actual biomass dry weights (*p* > 0.05). The linear regression model fitted well all data (averaged by treatment) for the total LiDAR scans compared with the vineyard dry biomass, indicating that the increasing number of scans corresponded to the increasing biomass values with an R^2^ = 0.85 (Figure 5). In addition, a positive linear fit was obtained for the comparison between actual dry biomass and LiDAR volume, both total volume and volume without tutoring prob, and both cases with an R^2^ = 0.75 (Figure 6).

**Figure 7:**
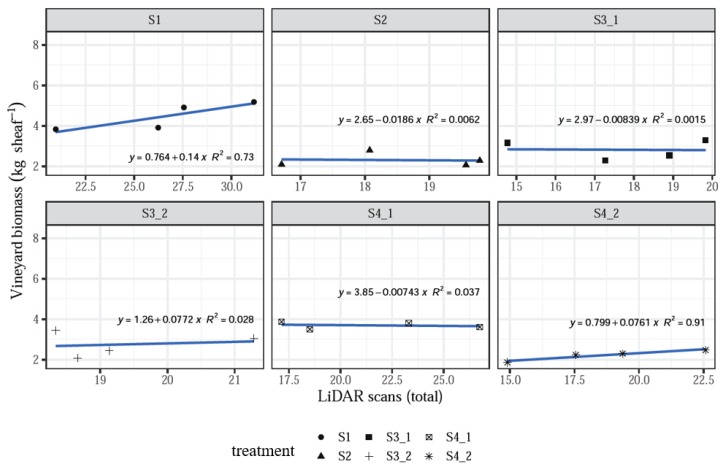
LiDAR scans versus vineyard biomass when analyzing data by treatment.

When analyzing data by treatment, it was found that the linear models fit well with some relationships where R^2^ > 0.60, while some treatments showed a poor correspondence of the LiDAR measurements and the actual biomass values, with R^2^ < 0.40 and even close to 0.006. Moreover, some relationships showed a negative slope, which does not correspond to the expected assessing function of the system, especially for treatments S2 and S4_1 (Figure 7).

However, the influence of individual treatments was insignificant (*p* > 0.1, Table 1), which still supports the hypothesis that increasing LiDAR measurement values correlate with increasing vineyard biomass measured in the field, as seen above with the data averaged by treatment (Figure 5 and Figure 6). The negative relationships may suggest that many more sampling plots should be acquired in order to satisfy the requirements to validate the system.

**Figure 8:**
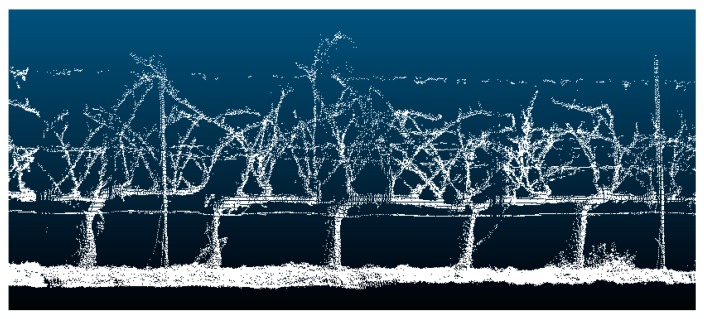
LiDAR data 3D point cloud. The crop row is divided into a 10-vine length. Note that training structures were rules out during branch volume calculation.

The high concordance between 3D structural information with actual parameters proved the accuracy of the method for evaluating pruning remains biomass using a non-contact sensor. Thus, the estimated branch volume was calculated accurately enough since the vine geometry reconstructed was of high fidelity (Figure 8).

Therefore, a high degree of agreement between branch biomass and volume was found. In accordance with the results of this methodology for volume estimation, hence biomass, it can be stated that the LiDAR sensor for capturing 3D geodata is a reliable remote-sensing method due to its capability to scan large crops in high geometric detail. High detail is necessary when a precision management decision needs to be taken. The majority of branches that should be pruned are of low diameter. The use of other systems such as depth cameras or low-cost photogrammetry methods are not able to capture such small detail. Thus, small branches are not properly reconstructed. Then, some management decisions (such as pruning) need to be made using high-resolution models created thought LiDAR systems or other high resolution devices. In this context, branch volume was calculated from computed point clouds from vines and indirectly estimated plant biomass (as a surrogate for pruning remains weight). The extracted volume values showed that the 3D models were able to properly estimate vine pruning weight in a fast and reliable manner. The obtained maps showed at high detail the spatial variation of the pruning weight along the vineyard rows. The created models can be of high value for the machinery developers and the wine industry. The low cost and fast assessment of the crop vigor can be translated to decision support systems. The information can be translated to automatic pruning systems or site-specific fertilization. In further studies, a higher computer performance is needed, since the data acquisition system was sometimes overloaded, resulting in scan loss; hence, some areas remained undersampled. This is due to the large datasets acquired by LiDAR sensors causing long processing times [19]. In addition, a R-squared value of 0.85 was found for the arithmetic mean of the number of scans and volumes. This could be explained by the training system used in the crop plantation. Although branches are relatively thin-stemmed, a vertical trellising helps vines to develop a regular and quasi-symmetric structure. This architectural structure allowed only one side of the crop to be scanned; thus, outliers would be minimized, and hence, they would have an insignificant effect on the volume calculation. Besides, there were no leaves helped as well in the regular plant geometry. This effect could lead to improvements in data processing, as branch biomass could be estimated indirectly only, considering the number of scans and the number of impacts of the laser beam. Measurements were taken from a defoliated vineyard, which is a major advantage to scan the vine structure, particularly vineshoots and cordons. Nonetheless, non-defoliated vines can affect the 3D model due to the effect of foliage occlusion, since the impossibility of measuring non-visible parts is an inherit limitation in any optical measurement [28].

Regardless of the crop stage, there are some sources of error that can be minimized such as the non-linearity of the mobile platform trajectory (uneven surface) along the crop alleys, since the sensor is free to rotate in three dimensions (roll, pitch, and yaw). As aforementioned, the LIDAR resolution is greatly affected by the vehicle speed, so it traveled throughout the readings at constant speed below 3 Km h-1, maintaining a steady course without maneuverings to follow a straight path. This is a key factor for obtaining accurate measurements [23]. Low speed in conjunction with its electric motor enables a vibration-free motion, which is highly convenient for high-quality information acquisition. Implementing an inertial measurement unit (IMU) would be a solution to encode angular displacements and correct the position of the LiDAR scans. del-Moral-Martínez, Rosell-Polo [29] corrected deviations using an IMU working at 100 Hz installed on a mobile terrestrial laser scanner to obtain LAI maps; however, the major source of error is linked to the distance between scans when overestimating LAI in areas where vines are less vigorous. However, some embodied errors in the LiDAR sensor, i.e., laser footprint (30.5 mm beam diameter in 1.5 m) and the accuracy of the kinematic mode (±20 mm) can also contribute to the propagation of uncertainty when reconstructing the crop architecture. A further alternative approach is to scan both sides of the chosen batches in order to improve vineshoot volume estimation. A fused GPS/IMU can help unify the scans in the same coordinate system; thereby, matching them with the scanning crop accuracy can be improved, as well as 3D reconstruction, hence making random errors negligible. 

The transition from traditional farming to digital solutions is demanded on several directives. A smarter, more modern, and sustainable agriculture is close. The agricultural policy must be opened up to research and innovation in support of the various roles played by agriculture and forestry by investing in technological development and digitization and by improving access to new knowledge. The use of a Farm Information System should be included in new farming procedures. It would lead to higher yields while minimizing the environmental impact of agriculture. Monitoring systems in combination with site-specific applications can reach these requirements. The use of monitoring procedures has shown the possibilities of reducing agrochemicals or minimizing water consumption. Application maps for site-specific application, variable rate, tillage, or pruning can be integrated on new machinery that is able to perform precise and differential treatments. Although the transition to Precision Farming is slow, a continuous evolution to the integration of new tools is demanded by farmers and consumers demanding safer and high-quality food. This methodology is a promising tool as a remote sensor with the capability for the non-destructive and accurate high-throughput measurement of branch volume. The integration on real fields can be easily done. However, consulting services and service companies could help with the adoption of this and other monitoring and site-specific management tools. Ongoing research is looking for improved tools for branch volume estimation, since the mobile platform can accommodate additional instruments. Moreover, a precise localization would be available from fused IMU/GNSS geodata to improve 3D reconstruction, as the two scanned sides (chosen vine batches) when georeferenced can be matched. The potential combination between LiDAR and other devices along with artificial intelligence could enable the development of new systems for estimating aboveground biomass that could feature stronger 3D models.

## 4. Conclusions

The current research is looking for new and improved systems for vineyard characterization. The presented method is of high accuracy and fast response at a low cost. The system consisted of a mobile platform equipped with a laser scanner, an RTK-GPS receiver, and an on-board computer. Three-dimensional (3D) models were possible, since the 2D LiDAR divide was displaced along the rows to obtain a 3D structure of the crop. The extracted volume values showed that the 3D models were able to properly estimate vine pruning weight in a fast and reliable manner. The number of scans by LiDAR influenced the relationship with the actual biomass measurements and had a significant effect on treatments. The models fitted well all data for the total LiDAR scans compared with the vineyard dry biomass, indicating that the increasing number of scans corresponded to the increasing biomass values. The high concordance between 3D structural information with actual parameters proved the accuracy of the method for evaluating pruning remains biomass using a non-contact sensor. However, some computational and point cloud processing issues still need to be improved. LIDAR technology has been proved for its capability for defoliated vineyard reconstruction with promising results. The low cost and fast assessment of the crop vigor can be translated to decision support systems. The information could be used for automatic pruning systems or site-specific fertilization. In addition, the information provided by the sensor could be utilized for a double purpose, not only for crop characterization. The distance measurements could be used in object detection and guidance for the improvement of the autonomous platforms.

## Figures and Tables

**Table 1:** Intercepts and estimates of the linear regression analysis by treatment with their corresponding standard error (SE) and significance levels (*p*-value).

Variable	Treatment	Estimate	SE	*p*-Value
Number	S1	0.1395	0.0635	0.0484
of scans	S2	−0.0186	0.1868	0.9224
	S3_1	−0.0084	0.1209	0.9458
	S3_2	0.0772	0.2017	0.7086
	S4_1	−0.0074	0.0606	0.9044
	S4_2	0.0762	0.0831	0.3773
Volume	S1	−45,105	54,263	0.4221
	S2	−23,180	23,265	0.3388
	S3_1	13,616	23,875	0.5799
	S3_2	−15,152	29,934	0.6219
	S4_1	−0.9339	16,265	0.5765
	S4_2	38,107	44,544	0.4090
Volume	S1	−42,186	55,154	0.4591
without	S2	−23,145	23,138	0.3369
prob	S3_1	14,104	23,863	0.5655
	S3_2	−16,114	30,194	0.6033
	S4_1	−0.9326	16,306	0.5779
	S4_2	38,107	44,617	0.4098
